# Cholera Infantum

**Published:** 1860-06

**Authors:** A. W. Griggs

**Affiliations:** West Point, Georgia


Cholera Infantum prevails so universally in the different portions of the United States, and is so fatal to the dearest interest of society, that we feel justified in presenting some views respecting the symptoms, cause and management of the disease. It prevails to a greater or less extent annually in our larger towns and cities, frequently visiting the less populated towns and villages, sweeping its hundreds to a premature grave. In and below this latitude it is common to see cases in the months of April and May, and in all the succeeding months until November, though if we have a late spring and an early winter, the disease is restricted to the summer. Children from four to twenty months old are most subject to it, yet both the older and those of a more tender age, frequently suffer from its effects. The period of first dentition is to be regarded as far the most obnoxious. At and during the *whole* of this time, and particularly according to my observation during the evolution of the *molar teeth*, there is a morbid *irritability* of the gastro enteric mucous membrane and a depraved *biliary* secretion, which, in connection with improper *diet* and a *hot* and confined atmosphere, is fully capable of developing the disease, with the alarming train of its subsequent symptoms. The relaxing influences of continued heat upon the general system, and its morbid effects in changing the secretory action of the liver, may be considered among the greatest of the predisposing, whilst the inflamed and congested state of the tissues of gums may be reckoned as the most common of the exciting causes. For the most part, the attack is after the following manner: the patient becomes preternaturally lax in its bowels, passing watery or jelly-like discharges for several days; is quite fretful, often asking or motioning for water, which, if allowed, either increases the number and aqueous character of the evacuations, or excites such inordinate emesis that the blandest ingesta will not be retained. There is usually great heat about the head and the mouth is sometimes very dry, especially the membrane covering the *gums*, at which the patient often pulls, frequently scratching it and its parched lips.

If there be no fever, the salivary secretion is considerable, in most cases, before the disease sets in, and the patient bites its fingers, and presses its gums against whatever is placed in its hands. If the case be permitted to take its own course, these symptoms grow more and more imminent, and the most rapid exhaustion ensues, which, but for the elasticity of the infantile constitution, would speedily result in death. (There seems to be a more enduring vitality in the system during the season of physical growth and development, than in maturity, and in this way we may account for the protracted symptoms of this disease, and the occasional recovery of the patient after weeks and months have elapsed with but litte abatement in their severity). Emaciation begins early, and increases until death relieves, or convalescence begins. The features are acute or pinched, the nose and lips thin, the latter being shriveled; the skin over the forehead tight and shining; the eyes glassy; pupils dilated; the orbits hollow. The eyes are half closed during sleep, and are so insensible that flies may crawl over the balls without producing any disturbance. The urine is usually scanty and red, occasionally otherwise. The tongue is white, and exhibits florid papillæ, upon its surface, which are paler and more elongated near the root. In the latter stages the coat is brown, and edges and tip somewhat red. The skin is generally dry, but moist at times, especially after vomiting; the extremities cool, and the head and abdomen hot. The pulse is feeble and frequent; the evacuations at first watery and of a greenish yellow—are changeable—assuming every possible morbid appearance as to color and character, excepting consistency. Most commonly they are quite green, mixed with mucous floculi, and are very fœtid and excoriating; they sometimes contain small quantities of blood, mixed with mucus, when the patient is much debilitated. They usually have an acid reaction, though not always, and I have known them to be altogether inodorous, or to emit a peculiar, faint, or fresh smell. The food is frequently passed from the bowels unchanged by the process of digestion. The patient often *picks* at its *nose*, and in the worst cases thrusts its fist far back into its mouth. It frets, twists its body with vermicular motion, and draws its knees toward the abdomen for several minutes before an action takes place, and louder complaint is made at the time.

Prolapsus ani is present in severe cases. The abdomen is very rarely swollen much, but is commonly lank and sunken, giving great prominence to the thorax. The stomach is highly irritable, rejecting alike food, medicine and water, which are retained but a few moments after being swallowed. Water, coffee and tea especially promote vomiting; raw milk is ejected in the form of hard cheesy flakes. In fact, in *many cases* vomiting is the *primary* symptom, and is attended with much thirst, the bowels being torpid. Usually in mild cases the matters ejected from the stomach, are such as have been allowed as nourishment, and including liquids to appease the thirst, but in more malignant cases, especially when the bowels are confined in the early stage, there is vomiting of bilious matter. Frequently the symptoms are those of ordinary cholera morbus, the vomiting and purging being both excessive and producing great prostration and langour in the intervals. In fatal cases, the vomiting, for the most part, continues throughout the attack, but generally it abates or ceases altogether as the irritation progresses to the lower bowels. Though these symptoms be permitted to go on unchecked, as exhausting as they are, the patient, in the majority of instances, outlives our expectations.

Marasmus sets in, and by slow but certain steps, leads the sufferer to the grave; or the brain becomes evidently affected—

the patient rolls its head from side to side, frequently throwing its body into a complete state of episthotonos; utters piteous moanings at short intervals; the conjunctiva is reddened by turgid and tortuous vessels; the eyes roll up, and coma and convulsions conclude the scene. I have known two cases in which the coats of the eye were ruptured and the humour discharged; this happened to my little boy, Edward Hill, who died on the 3d of June, 1851, aged 5 months and 19 days, and also to the child of a medical brother, I believe, on the succeeding year, which recovered with the loss of one eye.

A short time preceding death, an eruption resembling flea bites can be seen over the abdomen, and a slightly gangrenous odor is exhaled from the *skin*, which now assumes a dull, dirty cast. With the eruption I have often had the patient recover, but never with the last symptom.

Thus, I have, to the best of my ability and recollection, described what I have seen and read of this most fearful malady, and this has been done more to revive the medical mind in the remembrance of the great importance attached to the subject than any consideration of personal notoriety or advancement. We will now proceed to the treatment, which consists in removing the causes, or in modifying, to as great a degree as can be done, their action on the system; in establishing a healthy biliary secretion; a more normal action in the mucous follicles of the alimentary tube, and in controling the disordered sympathies of the various functions. The apartment of the patient should be thoroughly ventilated and well shaded, and in the cool of the day the patient should be carried into the open air, caution being taken against exposure to dampness. It would be best for the child to be removed to a purer and healthier atmosphere, but this is often impossible, on account of the indigency or the occupation of the parent. When the diarrhœa marks the beginning of the attack, the strictest hygienic regimen should be instituted. It would be improper to confine the bowels by the use of any remedies, as it would endanger the brain; a few portions of Calomel and Chalk, or Hydrarg C Creta will act well by promoting healthy secretion and controling acidity. The physician is rarely called so early as this, but receives his summons to treat the more formidable stage of emesis. This sometimes, especially if there is no great attraction in the secretions, is amenable to the action of simple measures, such as small quantities of beef tea given warm, while mint, steeped in hot brandy, is applied over the epigastrium. The mistura creta is a long tried and good remedy. A few portions of the carbonate of magnesia, with the addition of from one-half to two drops of laudanum, may prove serviceable, by carrying off offensive matter from the bowels and neutralizing acidity. Sinapisms to the epigastrium will add to the efficacy of the treatment. The gums should be freely scarified at once, and the operation repeated every two or three days, until the teeth make their appearance. Creosote, in doses from the eighth to the quarter of a drop, given in emulsion is highly useful both for controling gastric irritability and for its antiseptic properties. The sub-nitrate of Bismuth, in one or two grain doses, combined with an aromatic, has acted well in some cases. I have controled vomiting in all but hopeless cases by administering enemata of a solution of from two to five grains of the ext. calocynth c, to the f ℥ of warm water, the action doubtless due to the aloes entering into the composition, can readily be explained on the principle of exciting a distant local irritation, to overcome a disordered *sympathetic* irritability. I have, for six years, employed the following with the most flattering results:

℞.—Opii Pulv. j grs.; hydrarg chlorid mit, xij grs.; cretæ prep., xlviij grs.; cinnamomi pulv., xxiv grs.; misce bene, et fiant pulv., No. xvj.

*Sig.*—To a child from 12 to 20 months old, give one, in fine syrup, every four hours, until vomiting subsides and sleep is secured. For patients under 12 or over 20 months of age, the first ingredient must be diminished or increased, as the circumstances may require. During the treatment water must not be allowed except cold and in small quantities; a tea spoonful of ice water may be given every 40 or 50 minutes, or the patient may be permitted to suck a little ice occasionally to cool its burning gums. If water be allowed, *ad libitum*, it will be difficult, indeed impossible to control the symptoms, for it acts like poison in such cases, and only gratifies a morbid desire for a moment, afterwards to increase the irritability as well as the thirst. But few parents observe these things without their being pointed out, and many thus kill their off-spring inconsiderately, being moved by the promptings of the deepest affection. Occasionally the gastric and enteric irritation simultaneously give way to treatment, but the result is not so fortunate in the majority of instances, for diarrhœa commonly supervenes upon or continues after the cessation of vomiting, and sometimes an almost incredible number of evacuations take place during the day. I have known as many as thirty in twenty-four hours, all being small and slimy, or jelly-like, and highly offensive to the smell. Commonly, however, they are by no means so numerous. When very frequent, I am in the habit of using an enema every two or three hours, P. R. N., of the following:

℞.—Acacia, pulv., xv grs.; Aquæ, f ℥ iv grs.; Tannin, vj grs.; Tr. Opii, gtt. x to xx. Misce et Sig.

Inject into the rectum f ℥ ss. at a time, and apply to the anus a soft cloth saturated with ice water. Light red oak bark poultices may be applied to the abdomen, and a few tea-spoonsful of boiled milk, thickened with wheat flour, allowed occasionally. From one to two grains of hydrarg c creta should be given every fourth or sixth hour for its alterative effects, and if inflammatory action be absent we may beneficially alternate with some of the astringents. If there be but little fever, as is often the case, a little pure port wine, containing a few grains of tannin will not be inadmissible. When the stomach will bear it, the iron by hydrogen will be found useful in building up the deteriorated blood, and giving stamina to the muscular fibre. The usual acidity which is present in the first stage of the disease is generally removed by the plan previously given, and we have then a most potent medicine to control a diarrhœa mostly dependent on debility. I mean the Aromatic Sulphuric Acid. When mirasmus or inflammation of the brain ensue, they must be managed upon general principles.

Let us remember that we may often bring the disease under subjection and dismiss our patient before the danger is passed; for the symptoms are apt to return upon the subsequent eruption of any of the teeth. From exposure to the vicissitudes of temperature, and from thc use of such dietetic articles as may renew the irritation. *Caution* must be observed in reference to these things, for many times, indeed in nearly every case, after the more active symptoms have subsided, our great object is to carefully oppose everything capable of producing relapse until we can gain a safe harbor in the approach of the cold season.

There is but one thing more that I have to say, and that is in reference to clothing and diet. The person should be kept of an equable temperature, if practicable; this will be best effected by employing flannel slips of sufficient length to protect the extremities, to be worn next to the skin; the feet should be covered with woolen socks, and the head kept as cool as possible. The diet should consist exclusively of the mother's milk, if she be healthy and not pregnant; otherwise, a wet nurse of suitable qualities should be employed, or the milk of the cow, diluted with an equal quantity of water, boiled and thickened with wheat flour, or Bermuda arrow root may be substituted. In a few instances I have known the fresh milk undiluted, given warm from the animal, to answer well. The animal should be well treated, and the milk should be taken from the udder, strained, and given to the patient before the loss of its temperature. In the latter stages, the sweet potato, roasted in hot ashes, is an excellent diet, being slightly alkaline, astringent, unirritating and very nutritious. I have never witnessed any bad effects from its use. They possess the property of rendering the actions more consistent, which is no small item in the treatment.

